# The effect of adding colostrum or sodium butyrate to the diet on the intestinal barrier of weaned piglets

**DOI:** 10.1371/journal.pone.0342570

**Published:** 2026-02-25

**Authors:** Marek Pieszka, Kinga Szczepanik, Paweł Kubica, Maria Oczkowicz, Sylwia Orczewska-Dudek, Bogadan Śliwiński, Łukasz Gala

**Affiliations:** 1 National Research Institute of Animal Production, Department of Animal Nutrition and Feed Sciences, Balice, Poland; 2 Department of Analytical Chemistry, Chemical Faculty, Gdańsk University of Technology, Gdańsk, Poland; 3 National Research Institute of Animal Production, Department of Animal Molecular Biology, Balice, Poland; University of Illinois, UNITED STATES OF AMERICA

## Abstract

Weaning is a critical stage for piglets, often leading to intestinal barrier disruption, impaired nutrient absorption, and growth reduction. This study investigated the effects of dietary supplementation with dried bovine colostrum or sodium butyrate on intestinal barrier function, nutrient absorption, and growth performance in weaned piglets. Eighteen DanBred Hybrid piglets (28 days old) were allocated to three groups (n = 6): control (no additive), colostrum, and sodium butyrate. Piglets were fed standardized prestarter and starter diets for 28 days. Growth performance and feed conversion ratio (FCR) were monitored, and intestinal permeability was assessed using a sugar absorption test with LC-MS/MS analysis of urinary sugars. Additional evaluations included intestinal histomorphometry, brush border enzyme activities, immunohistochemistry for tight junction proteins, hematological and biochemical parameters, and gene expression analysis. Colostrum supplementation resulted in significantly higher final body weight and average daily gain compared with control and sodium butyrate groups (P < 0.001). FCR was consistently improved in the colostrum group during both feeding phases. Sugar absorption tests indicated greater urinary recovery of mannitol, lactulose, sucrose, and raffinose, suggesting enhanced intestinal permeability and nutrient uptake. Histological analysis showed longer jejunal villi and increased mucosal width (P < 0.01). Colostrum-fed piglets also exhibited higher sucrase and lactase activities and upregulated expression of occludin and claudin 5. Sodium butyrate produced positive but less pronounced effects, including increased claudin 1 expression in the ileum and dipeptidylpeptidase IV activity. Hematological parameters remained within reference ranges, with lower gamma-glutamyl transferase levels observed in the colostrum group, indicating reduced metabolic stress. No major differences were found in cytokine gene expression. In summary, spray-dried bovine colostrum supports gut health and metabolism in weaned piglets by enhancing intestinal barrier maturation. It boosts digestive enzyme activity and nutrient utilization, which contributes to improved growth. Sodium butyrate provided supportive but less consistent benefits. Overall, bovine colostrum represents a practical and effective nutritional strategy to improve the health and development of piglets after weaning.

## Introduction

The intestinal barrier is a functional zone of intestinal isolation that separates the intestinal tract from the internal environment of the body, preventing invasion, translocation of bacteria, and the penetration of allergenic compounds from the intestines into the body [1]. A damaged intestinal barrier or increased permeability in piglets is associated with stress related to weaning from the sow, social, environmental, and dietary stress due to feed change [2]. The gastrointestinal tract is an important organ of stress response in piglets, as it is the main site of digestion and nutrient absorption and an important line of defense against the invasion of bacteria and endotoxins into the intestinal lumen [3].

The stress associated with weaning also has a negative impact on intestinal development, physiology, microflora, and immunity [4,5]. Intestinal permeability is one of the main components of the intestinal barrier. This barrier is a dynamic connection between the body and food and pathogens that enter the digestive tract. Therefore, dietary components can directly influence this connection, and many metabolites produced by host enzymes or gut microbiota can act as signaling molecules or directly influence this barrier. The absorption of nutrients from the intestinal lumen occurs in two ways: transcellular and intercellular, through so-called tight junctions (TJ) [6,7]. Damage to or disruption of the normal function of tight junctions leads to increased and nonselective intestinal permeability. One of the most critical mechanisms is the establishment of a permeability barrier, which is mainly regulated by TJ, which consist of many intracellular and apical intercellular membrane proteins (e.g., zonula occludens, occludin, and claudins) [8]. Disruption of the intestinal barrier function underlies gastrointestinal and other systemic diseases such as food allergies, gastrointestinal infections, celiac disease, chronic inflammatory bowel disease, cystic fibrosis, rheumatoid arthritis, multiple organ injuries, alcoholism, stomach cancer, colon cancer, and Crohn’s disease [9–11]. It has been established that changes in intestinal permeability are one of the pathogenic factors in many gastrointestinal and systemic diseases [12]. Research on the possibility of stabilizing the intestinal barrier by reducing its permeability, both preventively against possible damage and after it has occurred, may offer the opportunity to apply new therapies or nutritional strategies. Increased intestinal permeability can lead to reduced nutrient absorption, resulting in decreased immunity and reduced production of digestive enzymes [13]. There are many metabolites produced by the enzymatic conversion of nutrients, either by host enzymes or by the gut microbiota, or by the stimulation of the release of non-enzymatic molecules that affect various functions, including changes in the intestinal barrier [14,15]. Metabolites produced in the lumen can enter the bloodstream and reach concentrations sufficient to affect the functions of the body’s organs [16]. A physiologically very important secretion of the mammary gland is colostrum, which influences the maturation of the gastrointestinal tract, the preparation of the immune system, and the development of beneficial microbiota [17–19]. These factors are relatively easy to isolate from colostrum or milk, but it is more difficult to provide evidence of their importance. The mere presence of a factor with a known effect in colostrum is not sufficient to assume an effect on the gastrointestinal tract [20]. To be physiologically relevant, a bioactive factor must pass through the gastrointestinal tract to its site of action without degradation. To facilitate this, colostrum contains glycoproteins and protease inhibitors (inhibiting trypsin, chymotrypsin, and elastase), and in addition, some factors are difficult to digest due to their acid resistance [21]. Conversely, some factors are activated by the acidic environment in the stomach, and bioactive peptides can be released from proteins during enzymatic digestion in the gastrointestinal tract [22–24]. Therefore, bioactive factors must be present in sufficient quantities, in an active state, and not be inhibited by other factors [21,25]. One of the most active substances affecting intestinal epithelial cells is butyrate, a short-chain fatty acid (SCFA) that is involved in several metabolic processes and has been extensively studied for its ability to improve intestinal function. Research on butyric acid has focused, among other things, on understanding the function of colonocyte nutrition, improving nutrient absorption, and in particular, rebuilding the epithelium damaged by intestinal inflammation, as well as supportive therapies for cancer and autoimmune intestinal diseases such as inflammatory bowel disease (IBD) [26]. In addition, butyrate can regulate the IL-10 receptor and the levels of zonulin, claudin, and occludin to reduce epithelial permeability and strengthen tight junctions [27,28]. The optimal functioning of these components is necessary for the proper function of the intestinal barrier, and disruption of the functioning of any of them leads to increased intestinal permeability and may cause various diseases. Despite growing interest in these substances, few studies have analysed their use in post-weaning piglets in the context of intestinal barrier function, and the results obtained are inconclusive. Therefore, assessing the effect of dried colostrum and sodium butyrate supplementation on intestinal epithelial integrity parameters is an important area of research. The research hypothesis assumed that supplementing the diet of weaned piglets with feed additives containing active substances would affect the integrity and intestinal barrier in young pigs.

The study aimed to evaluate the effect of adding dried bovine colostrum or sodium butyrate to the diet on the intestinal barrier in weaned piglets.

## Results

### Results of growth and feed intake parameters

The results concerning piglet body weight and feed intake and utilization are presented in Table 1. Supplementation of the piglet diet with bovine colostrum (group II) had a significant effect on the final body weight of piglets compared to the control group (I) and group III, which received sodium butyrate (P = 0.001). Significant differences were also found in piglet weight gains between weaning and 56 days of age between group II and the control group and experimental group III, respectively: 0.256 vs. 0.162 and 0.172 (P = 0.001). Significant differences were found between the groups of animals in the feed conversion ratio (FCR) in the prestarter period (P = 0.001), starter period (P = 0.002), and throughout the entire experiment, where significant differences were recorded between the groups, respectively: 1.321 (group II) vs. 2.128 (group I) and 1.829 (group III) kg/kg (P = 0.002).

**Table 1:** Effect of colostrum or sodium butyrate on zootechnical parameters.

		Mean				
		I	II	III	SEM	*P-*value
	Number of piglets, no	6	6	6		
Final weight, kg		12.57^de^	14.07^e^	11.97^d^	0.401	0.079
Average weight gain, kg	total	3.898^a^	6.142^b^	4.127^a^	0.270	<0.001
	daily	0.162^a^	0.256^b^	0.172^a^	0.011	<0.001
	total up to 42 days	1.872^a^	3.032^b^	1.943^a^	0.157	<0.001
	daily up to 42 days	0.117^a^	0.189^b^	0.121^a^	0.010	<0.001
	total from 43 to 56 days	2.027^a^	3.110^b^	2.183^a^	0.155	0.002
	daily for the period from 43 to 56 days	0.253^a^	0.389^b^	0.273^a^	0.019	0.002
Feed intake, kg	Prestarter	4.915	4.626	4.427	0.125	0.291
	Starter	3.330	3.503	3.068	0.094	0.163
	Total	8.245	8.129	7.495	0.854	0.275
FCR (kg/kg body weight)	FCR total	2.128^a^	1.321^b^	1.829^c^	0.087	<0.001
	FCR prestarter	2.690^a^	1.531^b^	2.357^a^	0.141	<0.001
	FCR starter	1.721^a^	1.129^b^	1.426^ab^	0.078	0.002

ADG – weight gain; ADFI – daily feed consumption; FCR – feed conversion ratio.

a,b – values in rows marked with different letters are statistically different at p ≤ 0.05.

### Results of hematological and biochemical analyses

Most of the results were within the reference ranges (Table 2, [29]). The number of white blood cells in group I was below the reference range (normal range 10–22 × 103/µL). A higher number of WBCs and LYMs was observed in group III compared to group I (P = 0.035, P = 0.039). The highest number of monocytes was in group III (P = 0.001). Statistically significant differences were noted for the other parameters (LYM%, MON%, GRA%, GRA, RBC, HGB, HCT, MCV, MCH, MCHC, RDWC, RDWS, PLT, MPV, PCT, PDW).

**Table 2:** Effect of colostrum or sodium butyrate on hematological and biochemical parameters of piglets.

	Nutrition group				
	I	II	III	SEM	*P-*value
Number of piglets, no	6	6	6		
Hematological parameters					
WBC, 10^3^/µL	8.189^a^	10.414^ab^	15.222^b^	1.18	0.035
LYM, %	58.619	57.403	57.778	1.19	0.922
MON, %	4.2	4.411	7.086	0.65	0.123
GRA, %	37.181	38.208	35.114	1.54	0.73
LYM, 10^3^/µL	4.736^a^	5.917^ab^	8.989^b^	0.73	0.039
MON, 10^3^/µL	0.289^a^	0.403^a^	1.097^b^	0.11	0.001
GRA, 10^3^/µL	3.172	4.108	5.136	0.43	0.18
RBC, 10^6^/µL	5.85	5.69	6.219	0.24	0.669
HGB, g/dL	9.739	9.286	9.900	0.36	0.789
HCT, %	32.492	33.775	34.747	1.65	0.869
MCV, µm^3^	59.769	59.647	58.847	0.68	0.853
MCH, pq	17.964	16.447	15.978	0.78	0.585
MCHC, g/dL	27.939	27.55	27.125	0.25	0.423
RDWC, %	17.983	17.864	18.353	0.24	0.707
RDWS, fl	37.264	37.781	38.453	0.42	0.547
PLT, 10^3^/µL	242.633	166.472	202.167	25.58	0.521
MPV, fl	7.85	7.794	7.844	0.09	0.963
PCT, ng/mL	0.163	0.131	0.159	0.02	0.802
PDW, %	35.486	39.339	40.306	2.03	0.619
Biochemical parameters					
TP, g/dL	4.702	3.877	3.885	0.27	0.378
GLU, mg/dL	110.750	109.833	92.750	5.26	0.308
CaARS, mg/dL	10.190	10.322	9.974	0.34	0.925
UREA, mg/dL	3.225	3.108	2.225	0.38	0.533
GGT, mg/dL	44.857^a^	33.452^ab^	17.539^b^	4.31	0.027
IRON, µg/dL	13.083	11.733	11.460	0.89	0.751
CRP, ng/ml	3.073	3.813	3.629	0.32	0.638
IgA, µg/ml	11.611	12.799	14.546	5.60	0.685
IgG, µg/ml	76.159	68.132	81.096	1.32	0.662

WBC – white blood cells, LYM (%) – lymphocyte percentage, MON (%) – monocyte percentage, GRA (%) – granulocyte percentage, LYM – lymphocytes, MON – monocytes, GRA – granulocytes, RBC – red blood cells, HGB – hemoglobin, HCT – hematocrit, MCV – mean corpuscular volume, MCH – mean corpuscular hemoglobin, MCHC – mean corpuscular hemoglobin concentration, RDWC – red blood cell distribution width, RDWS – red cell distribution width, PLT – platelets, MPV – mean platelet volume, PCT – plateletcrit, PDW – platelet distribution width, TP – total protein, GLU – glucose, CaARS – calcium (arsenazo method), UREA – urea, GGT – gamma-glutamyl transferase, IRON – iron, CRP – C-Reactive protein, IgA and IgG – immunoglobulin A and G.

a,b Values within a row with different superscripts differ significantly at P < 0.05.

Biochemical analyses showed higher GGT levels in group III compared to group I (P = 0.027). Other indices showed no statistical differences (TP, GLU, UREA, CAars, IRON). No statistical differences were found for CRP (ng/ml) and immunoglobulin A and G (ug/ml).

### Results of histomorphometry analyses

The results of the histomorphometry analysis are shown in Table 3. Histomorphometric analysis revealed no statistically significant differences in the duodenum. In the jejunum, group II exhibited a wider mucosa compared to group I (P = 0.004) and the longest villi (P = 0.005) of all groups. Several trends were observed: group III showed wider villi (P = 0.062), deeper (P = 0.074) and wider (P = 0.067) crypts than group I, while the villus length-to-crypt depth ratio was higher in group II than in group III (P = 0.088). No differences in villus width were found between groups. In the ileum, the narrowest crypts were observed in group I, with no significant differences detected in the other parameters.

**Table 3:** Results of histomorphometric analyses of piglet intestines.

	Nutrition group				
	I	II	III	SEM	*P-*value
Number of piglets, no	6	6	6		
duodenum					
width of muscularis, μ	348.630	462.534	564.798	43.09	0.168
width of mucosa, μ	541.166	617.032	553.780	24.25	0.416
lenght of villi, μ	308.068	319.376	306.021	13.00	0.913
width of villi, μ	132.337	126.245	131.432	4.17	0.831
depth of crypt, μ	266.272	341.737	286.355	16.29	0.147
width crypt, μ	39.509	41.066	40.032	0.78	0.733
ratio	1.271	1.041	1.167	0.09	0.624
jejunum					
width of muscularis, μ	397.647	319.646	419.315	23.44	0.194
width of mucosa, μ	411.427^a^	570.879^b^	497.406^ab^	19.29	0.004
length of villi, μ	286.093^a^	397.347^b^	309.560^a^	16.26	0.005
width of villi, μ	111.834^a^	124.225^ab^	125.161^b^	2.65	0.062
depth of crypt, μ	192.2723^a^	225.106^ab^	243.218^b^	9.45	0.074
width crypt, μ	36.298^a^	39.545^ab^	41.654^b^	0.97	0.067
ratio	1.573^ab^	1.886^a^	1.337^b^	0.10	0.088
ileum					
width of muscularis, μ	454.134	571.239	395.989	37.54	0.153
width of mucosa, μ	418.881	457.745	471.342	16.93	0.448
lenght of villi, μ	285.897	287.194	286.854	10.29	0.999
width of villi, μ	118.215	120.732	125.661	3.43	0.692
depth of crypt, μ	185.276	203.371	233.644	10.71	0.181
width crypt, μ	36.369^a^	40.933^b^	41.594^b^	0.78	0.003
ratio	1.653	1.475	1.358	0.07	0.259

I, II, III – group number, ratio – villium length to depth of crypts.

a,b Values within a row with different superscripts differ significantly at P < 0.05.

d,e Values in the row with different superscripts show a trend 0.05 < P < 0.1.

### Results of immunohistochemistry analyses

The results of the immunohistochemical analysis are shown in Table 4. Claudin 1 expression showed an upward trend in group III compared to group II (P = 0.060), with higher expression in the ileum of group III than in group I (P = 0.020). No significant differences were observed in the jejunal region. Claudin 5 expression was highest in the jejunum of group II and the ileum of group I (both P < 0.001). No significant differences were noted in the duodenal region. Occludin expression was highest in group II (P < 0.001), with no significant differences observed in the duodenal and ileal regions.

**Table 4:** Effect of colostrum or sodium butyrate on tight junction proteins in the intestine of piglets.

		Nutrition group			
		I	II	III	*P-value*
		Mean±SD			
Number of piglets, no		6	6	6	
	duo	1.122 ± 0.18^de^	1.093 ± 0.17^d^	1.295 ± 0.15^e^	0.060
CLDN1	jej	1.275 ± 0.33	1.072 ± 0.16	1.220 ± 0.36	0.520
	ile	1.107 ± 0.12^a^	1.259 ± 0.15^ab^	1.352 ± 0.15^b^	0.020
	duo	1.671 ± 0.28	1.704 ± 0.27	1.701 ± 0.32	0.919
CLDN5	jej	1.745 ± 0.25^a^	1.999 ± 0.18^b^	1.714 ± 0.36^a^	<0.001
	ile	1.735 ± 0.34^a^	1.521 ± 0.36^b^	1.392 ± 0.28^b^	<0.001
	duo	1.926 ± 0.23	1.810 ± 0.30	1.979 ± 0.45	0.095
OCLN	jej	1.377 ± 0.22^a^	1.834 ± 0.38^b^	1.339 ± 0.24^a^	<0.001
	ile	1.469 ± 0.30	1.369 ± 0.35	1.312 ± 0.21	0.092

I, II, III – group number, duo – duodenum, jej – jejunum, ile – ileum, OCLN – Occludin; CLDN1, 5 – claudin-1, 5.

a,b Values within a row with different superscripts differ significantly at *P* < 0.05.

d,e Values in the row with different superscripts show a trend 0.05 < *P* < 0.1.

### Results of genomic analyses

The genomic analysis results indicated a trend toward decreased expression of CLDN4 in the duodenum in group III compared to group I (P = 0.056), while the expression levels of the other genes (OCLN, IL6, MUC4, TGFB1, CLDN2, TNF, IL10, 1L4, CLDN1) showed no statistically significant differences between the groups (Table 5).

**Table 5:** Results of genomic analysis (qPCR) of piglet intestines.

	RQ			
	Mean± SD			
	I	II	III	*P-*value
Number of piglets, no	6	6	6	
Duodenum				
*OCLN*	1.138 ± 0.15	1.095 ± 0.08	1.144 ± 0.11	0.751
*CLDN1*	4.412 ± 7.85	1.107 ± 0.81	1.596 ± 1.19	0.878
*IL4*	1.943 ± 0.93	2.079 ± 0.56	1.720 ± 0.54	0.803
*IL6*	0.302 ± 0.36	0.157 ± 0.10	0.226 ± 0.09	0.554
*IL10*	0.644 ± 0.20	0.661 ± 0.14	0.683 ± 0.14	0.849
*TGFB*	1.087 ± 0.13	1.141 ± 0.14	1.081 ± 0.13	0.798
*TNF*	1.286 ± 0.26	1.503 ± 0.46	1.357 ± 0.34	0.683
Jejunum				
*OCLN*	0.953 ± 0.40	1.120 ± 0.34	1.024 ± 0.31	0.676
*CLDN1*	4.331 ± 6.39	7.013 ± 8.66	3.779 ± 2.88	0.523
*IL4*	1.298 ± 0.49	1.506 ± 0.61	1.456 ± 0.85	0.895
*IL6*	2.033 ± 2.39	2.945 ± 2.46	1.364 ± 0.57	0.676
*IL10*	1.255 ± 0.29	1.559 ± 0.21	1.355 ± 0.67	0.271
*TGFB*	1.062 ± 0.20	1.031 ± 0.12	0.880 ± 0.24	0.460
*TNF*	1.761 ± 0.93	2.037 ± 0.44	1.764 ± 0.64	0.309

I, II, III – group number, OCLN – Occludin; CLDN1, 2, 4 – claudin 1, 2, 4; IL4, 6, 10 – interleukin 4, 6, 10; TGFB – transforming growth factor beta; TNF – tumor necrosis factor.

d,e Values in the row with different superscripts show a trend 0.05 < P < 0.1.

### Results of activity of brush border enzymes the jejunum

In the proteins of the epithelium of the jejunum in the proximal, middle, and distal parts, the activities of the brush border enzymes: saccharase, lactase, maltase, aminopeptidase A and N, and dipeptidylpeptidase IV were shown in Table 6. Significantly higher saccharase activity was found in the initial and mid-part of the jejunum in the piglets in group II compared to the control group (I) and group III, respectively (P = 0.042 and P = 0.016). A significantly higher sucrase activity in the proximal and middle parts of the epithelium of the jejunum was also found in group II compared to groups I and III (P = 0.012).

**Table 6:** Activity of brush border enzymes of piglet jejunum (nM/min/mg protein).

		Nutrition group			
		I	II	III	*P-*value
	Number of piglets, no	6	6	6	
	Part of intestine	Mean± SD			
Saccharase	Prox	0.45 ± 0.08	0.48 ± 0.07	0.46 ± 0.08	0.121
	Mid	0.70 ± 0.10^a^	1.65 ± 0.09^b^	0.89 ± 0.07^a^	0.011
	Dist	0.30 ± 0.01	0.28 ± 01	0.26 ± 01	0.292
Lactase	Prox	10.54 ± 3.08^a^	20.4 ± 4.22^b^	11.96 ± 3.11^a^	0.040
	Mid	16.54 ± 1.60^a^	29.28 ± 1.98^b^	17.11 ± 1.66^a^	0.012
	Dist	11.12 ± 2.02	14.90 ± 2.09	10.86 ± 2.00	0.082
Maltase	Prox	7.22 ± 1.30	6.84 ± 1.20	7.48 ± 1.32	0.762
	Mid	4.94 ± 1.12	8.86 ± 1.28	6.91 ± 1.18	0.015
	Dist	3.02 ± 0.38	4.68 ± 0.41	4.98 ± 0.44	0.212
Dipeptidase IV	Prox	1.68 ± 0.50	1.86 ± 0.57	1.81 ± 1.55	0.114
	Mid	1.62 ± 0.44^a^	2.44 ± 0.52^b^	2.98 ± 0.56^b^	0.048
	Dist	2.02 ± 0.60	2.56 ± 0.64	2.88 ± 0.68	0.251
Aminopeptidase N	Prox	5.52 ± 1.46	6.42 ± 1.50	6.18 ± 1.49	0.068
	Mid	5.44 ± 1.68	6.00 ± 1.70	6.96 ± 1.74	0.056
	Dist	6.64 ± 1.60	7.44 ± 1.72	7.36 ± 1.68	0.092
Aminopeptidase A	Prox	6.50 ± .1.06	7.35 ± 1.16	7.12 ± 1.13	0.112
	Mid	4.80 ± 1.02	5.28 ± 1.06	5.68 ± 1.09	0.290
	Dist	3.84 ± 0.80	4.36 ± 0.86	5.02 ± 0.90	0.358

I, II, III – group number, Prox – proximal part of the jejunum, Mid – middle part of the jejunum, Dist -distal part of the jejunum.

d,e Values in the row with different superscripts show a trend 0.05 < P < 0.1.

### Results of chromatographic analysis of mannitol, lactulose, and sucrose content in pig urine using LC-MS/MS method

The results of the analysis of the content of mannitol, raffinose, lactulose, and sucralose in piglet urine are presented in Table 7. As a result of the sugar absorption test, piglet urine was analyzed for the content of mannitol, sucrose, lactulose, and raffinose using the LC-MS/MS method. A highly significant content of mannitol, raffinose, lactulose, and sucralose was found in group II receiving bovine colostrum supplement compared to group I (control) and group III supplemented with sodium butyrate, P = 0.001.

**Table 7:** Results of chromatographic analysis of the content of mannitol, sucrose, lactulose, and raffinose in pig urine using the LC-MS/MS method.

	Nutrition group			
	I	II	III	*P-value*
Number of piglets, no	6	6	6	
Saccharides	Mean±SD			
Mannitol, μg/mL	156.37 ± 9.05^a^	213.50 ± 8.30^b^	148.51 ± 6.77^a^	0.001
Sucrose, μg/mL	102.67 ± 2.77^a^	183.38 ± 5.68^b^	122.0 ± 3.36^a^	0.001
Lactulose, μg/mL	149.13 ± 3.58^a^	251.43 ± 6.85^b^	153.23 ± 5.82^a^	0.001
Raffinose, μg/mL	112.85 ± 2.22^a^	199.10 ± 1.67^b^	110.42 ± 1.46^a^	0.001

I, II, III – group number.

a,b Values within a row with different superscripts differ significantly at P < 0.05.

## Discussion

The pig’s gastrointestinal tract (GI) is one of the largest surfaces (400 m²) forming a barrier between the external and internal environments and plays a key role in regulating the immune system and, consequently, health [30,31]. The mucous membrane of the gastrointestinal tract performs the complex function of a semi-permeable barrier that allows the absorption of nutrients and immune sensitivity while limiting the transport of potentially harmful antigens and microorganisms. This seemingly “contradictory” task is regulated through the interaction of structural components and molecular interactions in the intestinal mucosa, which maintain intestinal integrity and immunohomeostasis [32]. Over 85% of passive transport occurs via intercellular pathways, so “tight junctions” are in practice responsible for its proper functioning. “Tight junctions” located on microvilli, on the intestinal lumen side, surrounding enterocytes in intercellular spaces, contain channels through which nutrients are passively absorbed. The number, density, size, and electrical charge of “tight junctions” determine the quantity and quality of absorbed substances [33]. In nature, weaning of pigs (wild boars) is a gradual process, completed at around 10–12 weeks of age, coinciding with the almost complete maturation of the gastrointestinal tract epithelium, immune system, and nervous system. However, in industrial pig production, weaning occurs suddenly between 14 and 30 days of age. An important stress factor for piglets is the moment of weaning from the sow, compounded by additional psychosocial and immunological stressors that increase stress during this period, including transport, mixing, fighting and establishing a new social hierarchy, vaccinations, etc. The moment of commercial weaning also coincides with the period of decline in passive immunity derived from the sow’s milk, which puts additional strain on the piglet.

In this experiment, slight leukopenia was observed in piglets from the control group. According to some studies, early weaning reduces the total number of leukocytes and weakens cellular immunity [34] Both supplements tested increased the WBC count to within the reference range, particularly bovine colostrum. In newborn animals, gamma-glutamyl transferase (GGT) serves as an indicator of colostrum intake: its blood concentration rises rapidly following ingestion of maternal colostrum. Elevated levels in neonates reflect absorption of this enzyme from colostrum. In piglets, GGT activity increases markedly after colostrum intake and then declines to adult levels within about one week. Beyond the neonatal period, the role of GGT shifts—it becomes a marker of liver function and metabolic stress. Weaning represents a significant stressor for piglets, resulting in intestinal and metabolic disturbances. Under such stressful conditions, including weaning, blood GGT activity tends to increase [35,36]. This study noted a significant decrease in GGT in piglets receiving bovine colostrum (the value was still within the reference range). It can therefore be assumed that colostrum played a protective role against excessive stress resulting, among other things, from the weaning process. In response to oxidative stress associated with weaning, the activity of superoxide dismutase and glutathione peroxidase increases [37]. Bovine colostrum contains a variety of enzymatic and non-enzymatic antioxidants, including superoxide dismutase and glutathione peroxidase [38]. Therefore, supplementation with bovine colostrum could alleviate the stress associated with weaning piglets from sows by increasing their total antioxidant defence. In addition, the main types of growth factors present in bovine colostrum, such as epidermal growth factor (EGF), platelet-derived growth factor 9 (TGF-β) and IGF [39], may have stimulated intestinal tissue growth, improving absorption capacity [40], which may also explain the observed benefits in terms of improved weight gain in piglets. Weaned piglets can survive and overcome the stress of weaning; however, it is essential to recognize that early weaning stressors occur during a critical period of digestive barrier development [41]. Most of the data on ways to protect and prevent damage to the intestinal barrier comes from experimental work on animal intestines and cell cultures [42,43]. A new approach to the problem of bacterial infections in piglets is to search for nutritional solutions that would interfere as little as possible with the structure of the intestines, especially the epithelium, where the action of such an agent consists in stabilizing the composition of the intestinal microflora, creating the most favorable conditions for digestion and absorption of nutrients.

There are many known factors that directly or indirectly lead to increased intestinal permeability. Undoubtedly, the most common among them are infectious agents (viruses, bacteria, and parasitic infections of the gastrointestinal tract). In this experiment, we examined the effect of administering sodium butyrate or bovine colostrum to piglets after weaning on selected elements of the intestinal barrier. In our experiment, we found a significant increase in small intestine permeability in piglets receiving bovine colostrum, which may indicate faster maturation of the intestinal epithelium structures in these animals and more efficient functioning of the intestinal barrier. The rapid development and proper maturation of the gastrointestinal tract, especially the immune system, at a young age in piglets seems to be a positive phenomenon, promoting rapid growth and development of the intestinal epithelium and ensuring the animal’s health [11]. In piglets, the period after weaning is associated with a decrease in feed intake, which leads to changes in intestinal morphology. Efficient regeneration of the gastrointestinal mucosa is crucial for piglets to quickly return to full health. In the described experiment, changes in the histomorphometry of the jejunum and ileum were observed under the influence of the tested additives. The addition of bovine colostrum improved villus length and mucosal width, which may have promoted increased nutrient absorption. Similar results were obtained by Rasmussen et al. (2016 [44]) in a study on piglets. The colostrum supplement influenced, among other things, the lengthening of villi and the improvement of the villi:crypt ratio compared to feeding with donor milk or milk formula. Similarly, a study by Mei et al. (2006 [45] showed that the height of intestinal villi and the depth of crypts were significantly greater in piglets fed pig or bovine colostrum than in piglets fed pig milk, modified milk, or water. This may therefore suggest that bovine colostrum can promote mucosal growth, which in turn may lead to improved absorption efficiency. In turn, studies on the effect of sodium butyrate show an ambiguous impact on the histometric parameters of the small intestine. In the study by Biagi et al. (2007 [46]), no significant differences in intestinal morphology were found after adding butyrate to the diet of piglets. In contrast, Kotunia et al. (2004 [47]) reported a decrease in villus length in the duodenum and an increase in villus length in the jejunum and ileum after adding butyrate to the diet of newborn piglets. Research by Wang et al. (2005 [48]) showed an elongation of intestinal villi under the influence of this additive. Lu et al. (2008 [49]) reported that sodium butyrate supplementation improved intestinal morphology in a dosedependent manner: 500 mg/kg increased villus height and the villus height–to–crypt depth ratio in the jejunum vs. control, while 1000 mg/kg further enhanced these parameters in the small intestine overall, as well as in the jejunum and ileum, compared to both control and 500 mg/kg groups. It can therefore be assumed that bovine colostrum has greater and more predictable potential in stimulating the regeneration of the intestinal mucosa in piglets after weaning, while sodium butyrate may play a supporting role, but requires optimization of the dose and method of administration. The study showed that the nutritional supplements used differentiated the expression of tight junction proteins (claudin 1, claudin 5, occludin) in a manner dependent on the intestinal segment. The most beneficial changes in terms of improving intestinal barrier tightness were observed in the group receiving bovine colostrum (for occludin and claudin 5) and in the group receiving sodium butyrate (for claudin 1). Similar results were obtained by Wang et al. (2012 [27]) on a monolayer of cdx2-IEC epithelial cells grown on Transwell filters. It was found that sodium butyrate improved the functioning of the small intestine barrier by increasing the transcription of the TJ protein claudin-1, facilitating the interaction of its promoter with SP1. Roselli et al. (2007 [50]) demonstrated that three fractions of bovine colostrum have the ability to protect IPEC-1 cells against increased permeability induced by ETEC. Despite differences in immunoglobulin and growth factor content, all fractions exhibited comparable protective effects, indicating that these components are not the main determinants of the observed effect. Compounds with antimicrobial properties, such as lactoferrin, lysozyme, and lactoperoxidase, as well as oligosaccharides and glycoproteins that can modulate bacterial adhesion to host cells [51,52], are considered potential mediators of this activity. The level of expression of pro-inflammatory and anti-inflammatory cytokine genes and genes contributing to the construction of the intestinal barrier is important for the health of weaned animals. In the genomic analysis, we did not observe any statistically significant differences in the analyzed genes: OCLN, IL6, MUC4, TGFB1, TNF, IL10, IL4, CLDN1. The lack of significant differences in mRNA levels for most of the analyzed genes contrasts with immunohistochemical results, which indicated segmental changes in the expression of tight junction proteins (e.g., changes in CLDN1 or OCLN). This may be due to changes during translation. The observed discrepancy between mRNA levels and protein expression for genes such as CLDN1 and OCLN may result from post-transcriptional and post-translational regulation. Transcript stability and translation are modulated by, among other things, microRNAs, RNA-binding proteins, and chemical modifications such as m⁶A methylation, which can reduce protein synthesis without altering mRNA expression. In addition, post-translational modifications of tight junction proteins — including phosphorylation, ubiquitination and glycosylation — affect their location in the membrane, half-life and interactions with the cytoskeleton, which may lead to segmental distribution disorders regardless of transcript levels. This is consistent with observations that the homeostasis and function of tight junctions are particularly sensitive to inflammatory signals and environmental stress [53,54]. Referring to the problem of nutrient absorption. One of the promising ingredients that helps alleviate inflammation of the intestinal epithelium is bovine colostrum, which contains numerous leukocytes (white blood cells) derived from the maternal circulation: lymphocytes, monocytes, macrophages, and neutrophils, which protect the newborn against infectious agents, including through secreted cytokines and the process of phagocytosis. They also regulate the development of the infant’s immune system. Among the growth factors in bovine colostrum, IGF-I and IGF-II predominate, as in human colostrum. By binding to specific receptors on the cells of the gastrointestinal mucosa, they have an anabolic effect, i.e., they promote their proliferation and differentiation. They modulate the tightness of tight junctions between enterocytes, thereby regulating the permeability of the intestinal epithelium.

In our study, piglets receiving bovine colostrum showed greater intestinal permeability in the SAT test, which was reflected in their higher body weight gains. This may indicate more intensive transport of nutrients into the bloodstream and other tissues. The increased concentration of indicator sugars in urine—mannitol, sucrose, lactulose, and raffinose—suggests enhanced permeability across all sections of the small intestine, pointing to dynamic development of the intestinal epithelium and accelerated organismal growth. Factors such as higher water intake or physiological changes associated with intensive post-weaning development may also contribute to the elevated sugar concentrations [55]. These findings are consistent with earlier observations by Kalach et al. (2001 [56]), who reported an initial increase, followed by a decline, in intestinal permeability in infants up to approximately three months of age, reflecting gradual maturation of the intestinal barrier. Complementary differences in the morphology of the gastrointestinal tract further support this interpretation. Piglets from the colostrum group were larger, had higher body weight, and exhibited a longer small intestine (unpublished data), which undoubtedly increased the total absorptive surface area. A larger absorption area allows a greater amount of marker sugars, including mannitol, to pass through the intestinal barrier and into urine, thereby indicating higher permeability. Thus, it appears that the enhanced permeability observed in colostrum-fed piglets resulted primarily from greater intestinal length and absorptive capacity rather than from alterations in epithelial structure. Importantly, our results confirmed that the addition of bovine colostrum to the diet of weaned piglets did not induce any negative changes in the membrane structures of the intestinal epithelium, including TJ.

Supplementing the diet of piglets with bovine colostrum significantly affected the final weight of piglets compared to piglets that were not supplemented and received sodium butyrate, which was reflected in weight gains, which translated into a reduction in the FCR index in animals whose diet was enriched with bovine colostrum, which corresponds to the results of other researchers, Boudry et al. (2008 [57]). However, as in our experiment, Sadurní et al. (2023 [58]) did not confirm a significant improvement in weight gain and a reduction in feed consumption. Studies clearly indicate that diet is the most important factor influencing the development of the digestive system, especially the reorganization of the small intestine mucosa. Any adverse changes in the composition of the food fed to newborns disrupt the normal development of the digestive tract and the entire organism [59]. The main source of energy for colonocytes are short-chain fatty acids (butyric, acetic, and propionic). They are produced as a result of bacterial fermentation of fiber in the intestines. Scientific studies have confirmed an improvement in intestinal barrier function after the use of psyllium and oats [60]. Butyrate, a four-carbon short-chain fatty acid (SCFA) and one of the most important end products of bacterial fermentation, deserves special attention in nutrition. Butyrate has a number of beneficial effects on the intestinal epithelium that extend far beyond the colon. Butyrate serves as an energy source or “fuel” for epithelial cells, has a trophic effect on the intestinal mucosa, reduces intestinal inflammation, strengthens the intestinal barrier function, and stimulates the secretion of pancreatic and jejunal enzymes. In addition, butyrate modifies the microbiota of the ileum and facilitates apoptosis of colon cancer cells [61,62]. There is also evidence that SCFAs may have a strong influence on the characteristics of gastrointestinal contractions. Increased colonic muscle contractility in response to butyrate was demonstrated by Soret et al. (2010 [63]) in both in vitro and ex vivo studies in rats. On the other hand, reduced contractile activity has been reported in the colon muscles of rats after consumption of butylated starch [64], as well as in the stomachs of pigs after infusion of butyric acid into the ileum [65]. Feeding newborns with artificial milk formulas delays the maturation of the gastrointestinal mucosa. In studies by Kotunia et al. (2004 [47]), sodium butyrate supplementation in newborn piglets did not affect the activity of brush border enzymes in the intestines. However, in our experiment with weaned piglets receiving bovine colostrum supplementation, we found significantly higher lactase activity in the initial and middle sections of the small intestine in piglets after weaning. The results of a series of studies indicate that the activity of lactase, an enzyme of the brush border, remains high in piglets during the suckling period. After weaning, its activity decreases significantly. In our experiment, we found higher activity in group II. This was due to the fact that piglets in group II received colostrum supplements (20 g/day/piglet). The lactose contained in the diet was probably the reason for the increased activity in this group of animals [66]. This indicates the stimulating and sustaining effect of colostrum, which is consistent with the results of Marion et al. (2005 [67]), although most authors indicate a decrease in the activity of this enzyme [68,69]. We can assume that the addition of dried bovine colostrum to the diet had a beneficial effect on the development of the intestines, including the muscular and mucous membranes of the duodenum and small intestine, where brush border enzymes are synthesized [70]. In addition, the relatively high proportion of whole milk in the mixture (16%) for piglets may have affected lactase activity. Furthermore, significantly higher dipeptidase IV activity was found in piglets supplemented with colostrum and butyrate compared to the control group. Our results indicate that substances that stimulate the development of the intestinal epithelium, such as butyrate or dried bovine colostrum, affect the activity of certain brush border enzymes [71]. We can conclude that the effects of weaning on intestinal enzyme activity appear to be dependent on the age at weaning. Such age-dependent differences in brush border enzyme activity may result from changes in the rate of cell renewal (crypt cell proliferation, epithelial cell migration from crypts, villus cell apoptosis) and protein synthesis (gene expression, protein maturation, and stability) [67].

In conclusion, supplementation with spray-dried bovine colostrum effectively supports intestinal health and metabolism in weaned piglets by alleviating weaning stress and improving the maturation and function of the intestinal barrier. Colostrum stimulates digestive enzyme activity, enhances immune function, and promotes better nutrient utilization, which translates into improved growth performance.

Sodium butyrate showed supportive effects, although less consistent than those of colostrum. Overall, the results indicate that bovine colostrum is an effective and practical nutritional strategy for improving the health and development of piglets after weaning.

## Materials and methods

The experiment was conducted in the animal house of the Department of Animal Nutrition and Feed Science at the National Research Institute of Animal Production. In accordance with Polish law and European Union Directive 2010/63/EU, the study conducted as part of this research did not require approval from the Local Ethics Committee for Animal Experiments. All procedures complied with relevant guidelines concerning animal experimentation and the care of animals used in research. No procedures involving pain or suffering were performed. All analyses were carried out using post-mortem samples, and the slaughtering of animals solely for the purpose of obtaining organs or tissues is not classified as a procedure under the directive.

### Animals, housing, diet, and experimental design

**Fig 1:**
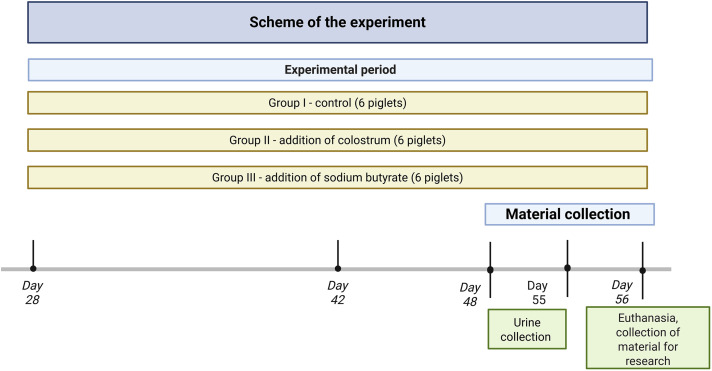
Schematic of the experiment.

The experiment was conducted on 18 boars (2 × 9 piglets each) of the DanBred Hybrid breed at 28 days of age. The piglets were weighed on a laboratory scale to the nearest 0.01 kg and labeled. Their average initial piglet weight was 8.14 ± 0.33 kg. The piglets were then randomly assigned to 3 feeding groups (Fig 1), with 6 individuals in each (n = 6). Piglets were placed in metabolic cages, one in each, and maintained under controlled environmental conditions at 20°C, 60% humidity with a 12-hour light/dark cycle. The cages were provided with feeders and teat feeders, and the animals were fed an amount of 2% of their body weight. Feed was fed twice at 7 a.m. and at 3 p.m. From day 28–43, piglets received a weaning prestarter mix, and from day 43 to the end of the experiment, a starter mix (Table 8).

**Table 8:** Composition of compound feeds.

Composition of weaning feed	Prestarter	Starter
Barley, %	22,7	22,0
Wheat, %	20,0	27,0
Corn, %	15,0	20,0
Post-extraction soybean meal, %	25,0	21,0
Casein, %	9,0	4,50
Soybean oil, %	3,0	2,0
Forage chalk, %	0,8	0,80
1-Ca Phosphate, %	1,10	1,10
Feed salt, %	0,18	0,20
L-lysine, %	0,40	0,48
DL-methionine, %	0,15	0,20
L-threonine, %	0,12	0,15
L-Tryptophan 98%	0,05	0,07
Premix^1^, %	0,5	0,5
Nutrient content per kg DM:		
Metabolic energy, MJ	14,2	13,8
Crude fiber, g	30	40
Crude protein, g	200	180
Crude fat, g	47,5	42,8
Lysine, g	13,0	12,0
Methionine+cystine, g	6,6	6,5
Threonine, g	8,0	7,6
Tryptophan, g	2,5	2,2
Calcium, g	7,6	7,6
Phosphorus, g	6,5	6,0
Sodium, g	1,6	2,0

^1^Composition of LNB’s mineral and vitamin premix in 1 kg: Na – 50 g, Ca – 208 g, P – 42 g; Mg – 10 g; Lysine – 60 g; Methionine – 12 g; Valine – 10 g, Threonine – 21 g, Vitamin A – 380000 IU; Vitamin D3 - 50000 IU; Vitamin E – 3500 mg; Vitamin K3 - 125 mg; Vitamin B1 - 57 mg; Vitamin B2 - 152 mg; Vitamin B3 - 1000 mg; Vitamin B6 - 114 mg; Vitamin B12 - 1.2 mg; Niacin – 1000 mg; Folic acid – 125 mg; Biotin – 7.5 mg; Fe – 3300 mg; Mn – 1330 mg; I – 50 mg; Zn – 3010 mg; Cu – 510 mg; Co – 40 mg; Se – 12 mg.

The mixtures were prepared according to the standards of Grela et al. (2020 [72]). From the beginning of the study, i.e., from day 28, the following additives were fed with the feed: group I, control – no additive, group II – dried colostrum (colostrum), and group III – sodium butyrate, in the amounts shown in Table 9. The dosage of the agents was based on scientific publications demonstrating the effect of the tested additives on animals [73,74]. Spray-dried bovine colostrum used in this study was sourced from COLOSTRUM POLSKA (Poland). Sodium butyrate was administered in the form of Adimix® Precision – Butyrate for Poultry & Swine (Poland), a preparation protected by palm oil salts.

**Table 9:** Dosing of feed additives.

Type of supplement	Dosage of the supplement, grams/piglet		
	Group I	Group II	Group III
Bovine colostrum, g	0	20	0
Sodium butyrate, g	0	0	1,0

The amount of feed intake was monitored daily. At weekly intervals, animals were weighed on a laboratory scale with an accuracy of 0.01 kg. The following zootechnical data were collected throughout the experiment:

- piglet weight, average weight gain
- average daily feed intake (ADFI) was calculated for each litter separately and duration according to the formula:

$$𝐀𝐃𝐅𝐈=\frac{\text{amount of feed taken per litter per animal per period}[\text{kg}]}{\text{duration of the rearing period}}$$

- feed conversion ratio (FCR): was calculated separately for each animal and the length of the fattening period according to the formula:

$$𝐅𝐂𝐑\;=\frac{\text{feed consumption per rearing period }[\text{kg}}{\text{total weight gain per rearing period }[\text{kg}]\;}$$

During the relevant experimental period, a sugar absorption test and urine collection were carried out on days 48 and 55 of the piglets’ lives. On day 56, the animals were killed using the standard method by stunning with a specialized Blitz penetration device (Germany), along with 9 × 17 mm caliber cartridges designed for pig slaughter. Blood, fecal, and tissue samples were collected for further analysis (stomach pyloric part, duodenum, jejunum, and ileum).

### Hematological and biochemical analyses

At animal slaughter, blood was collected into tubes with EDTA-K3 (Equimed, Poland) and then immediately analyzed for basic hematological blood terms, including white blood cells, red blood cells, hemoglobin, hematocrit, mean corpuscular volume, mean corpuscular hemoglobin, platelets, red blood cell distribution width, mean platelet volume, and mean corpuscular hemoglobin concentration. The second portion of the blood was collected into test tubes with a clotting activator (Equimed, Poland), centrifuged on a laboratory centrifuge for 15 minutes and 4500 rpm, and the obtained serum was transferred to sterile Eppendorf-type tubes and placed at – 80°C until analysis in the laboratory. Analyses of selected biochemical terms, including UREA, TP, CREA, CaARS, IRON, and GGT, were performed by spectrophotometric method on a Mindray BS-180 automatic analyzer (Shenzhen Mindray Bio-medical Electronics Co. Ltd., Shenzhen, China), using dedicated diagnostic kits from PZ Cormay SA (Warsaw, Poland) following the manufacturer’s instructions. C-reactive protein, immunoglobulin A, and immunoglobulin G analyses were performed by the ELISA method using ELISA Diagnostic Kits (BT-Lab, China), and measurements were made on an ELISA reader, Synergy H1 Multimode Reader (BioTek, United States). For each individual, 2 measurements were taken, from which the average was calculated.

### Histological analysis

Samples of the small intestinal segments (duodenum, jejunum, and ileum) and stomach (pyloric part) were obtained and processed for histological analysis. Briefly, a section of the duodenum, jejunum (taken halfway along its length), and ileum (taken 2 cm before the ileocecal junction), stomach, and liver were rinsed with saline solution and placed in buffered formaldehyde solution (4%). After 18–24 h, the fragments were cut into approximately 0.5 cm sections and placed in histology cassettes, followed by dehydration in graded ethanol solutions (up to 70%). The cassettes were placed in a tissue processor, where a cycle of transitions in ethanol (Idalia), non-polar solvent (Xylene; Idalia), and paraffin (Histoplast; Thermo Shandon Limited) permeabilization was set. Two slides were made from each paraffin block, and two slides were made; the blocks were cut using a microtome (Microm HM 340 E; Thermo Scientific) into 4 μm sections. The slides were stained by a standard method using Harris hematoxylin and alcohol eosin, and then sealed using Consul Mount histological glue (Elektromed, Poland) and a coverslip. In each histological preparation, 15–20 measurements of each studied parameter were taken. Photographs and subsequent measurements were taken using an Olympus BX63 automated microscope (Olympus, Germany) equipped with a DP74 camera with accompanying graphic image analysis software (CellSens, Olympus). For the intestines, parameters such as the height and width of the intestinal villi, the depth and width of the crypts, the thickness of the mucosa, and the muscularis mucosa were evaluated, and the ratio of the length of the villi to the depth of the crypts was calculated. For the stomach, the width of the mucosa and muscularis were measured.

### Immunohistochemical analyses

The tight junction proteins claudin 1, 5, and occludin were evaluated in intestinal tissues. Tissues of the duodenum, jejunum, and ileum were placed on adherent slides. Immunohistochemical staining for claudin 1, claudin 5, and occludin was performed after deparaffinization in xylene and rehydration with reduced concentrations of ethanol and distilled water. Heat induced epitope recovery was performed in sodium citrate buffer (10 mM sodium citrate, pH 6.0) using a laboratory microwave. The sections were then cooled to room temperature and washed with Tris buffered saline (TBS) buffer. The slides were then incubated in an antibody blocking solution (UltraCruz® Blocking Reagent; Santa Cruz Biotechnology) for 40 min at room temperature. To block nonspecific binding, the slides were incubated in 5% goat serum solution (ab7481; Abcam) for 30 min at room temperature. The slides were washed with TBS buffer. The slides were then incubated with primary antibodies claudin 1 (AF0127; Affinity), claudin 5 (AF5216; Affinity), and occludin (DF7504; Affinity) for 1 h at room temperature in a humid chamber. The slides were washed with TBS. The preparations were then incubated with Goat Anti-Rabbit IgG (Alexa Fluor® 594; abcam ab150092) for 30 min at room temperature. After the slides were washed in TBS, they were sealed with VECTASHIELD® PLUS Antifade Mounting Medium with DAPI (Biokom, Polska). The latter was used to image cell nuclei. Immunoreaction was verified with negative control subjected to identical immunohistochemical staining, excluding the use of primary antibody. Photographs and subsequent measurements were taken using an Olympus BX63 automated microscope (Olympus, Germany) equipped with a DP74 camera with accompanying graphic image analysis software (CellSens, Olympus). Imaging measurement analysis was performed using ImageJ (version 1.53; US National Institutes of Health). The analysis tool in Image-J was calibrated with Kodak StepTablet to measure optical density by the Rodbard method. The tablet has 21 steps with a density range of 0.05–3.05 optical density. The intensity of immunoreaction was measured in ten randomly selected areas for each animal. From the results obtained, an average value was taken for statistical analysis. The area of interest included only a section of the epithelium, significantly reducing the background’s effect on the measurement. For immunofluorescence, the lower the measurement value of the calibrated image, the higher the protein expression, and vice versa.

### Sugar absorption assay – chromatographic analysis of: mannitol, lactulose, raffinose, and sucrose in porcine urine by HPLC

After 12 hours of starvation, pigs—having been on a diet devoid of lactulose, mannitol, sucralose, and raffinose for 24 hours prior—were given a solution of 2 g mannitol, 10 g lactulose, 10 g raffinose, 20 g sucrose, and 20 g sucralose in 250 mL deionized water; baseline (clean) urine was collected before dosing to correct for endogenous carbohydrate presence, then urine was collected over the next 5 hours (with each pig receiving 250 mL of clean water after the first 2 hours), and 0.1 mL of 1% chlorhexidine aqueous solution was added to each urine dish as an antimicrobial agent; collected samples were immediately frozen at –20 °C. For chromatographic analysis, analytical standards of lactulose, mannitol, sucrose, and raffinose, LC-MSgrade acetonitrile, ammonium formate, Amberlite MB20 ion-exchange resin, and 13 mm, 0.22 µm nylon syringe filters were purchased from Merck (Warsaw, Poland), and ultrapure water was prepared using an HLP5 system (Hydrolab, Wiślina, Poland); the method was modified based on Kubica et al. (2012). Samples were stored at –80 °C until analysis, at which time they were thawed to room temperature; 1 mL of urine, 1 mL of ultrapure water, and 50 mg of ion-exchange resin were added to a 10 mL glass test tube and mixed for 5 minutes at 800 rpm, then 200 µL of supernatant was transferred to a clean tube, mixed with 800 µL acetonitrile, filtered through a 0.22 µm, 13 mm nylon syringe filter, and transferred to a chromatographic vial for LC-MS/MS analysis. Calibration standards for each analyte covered 1–25 µg/mL (1, 2, 5, 10, 15, and 25 µg/mL) and were prepared and analyzed in triplicate (n = 3). All analyses were performed on a Shimadzu LCMS-8060 triple quadrupole LC-MS/MS system (Japan) equipped with an electrospray ionization source operating in negative-ion multiple reaction monitoring (M-H) mode; optimal detection conditions for each analyte are provided in Table SM2. Chromatographic separation was performed using a Nexera X2 ultra-performance liquid chromatography (UPLC) system (Shimadzu, Japan), comprising a DGU-20A5R degasser, CBM-20A system controller, two LC-30AD binary pumps, an SIL-30 AC autosampler, and a CTO-20 AC column oven. Separation was achieved using a Phenomenex BioZen Glycan column (2.6 µm, 100 Å, 100 mm × 2.1 mm) equipped with a guard column. The mobile phase was maintained at a constant flow rate of 0.9 mL/min throughout the analysis, with an injection volume of 5.0 µL. The mobile phase consisted of (A) 20 mM ammonium formate buffer, pH 6.8 (20%), and (B) acetonitrile (80%). The analysis was conducted under isocratic elution conditions with 20% A and 80% B. The total analysis time was 4 minutes. The column temperature was maintained at 45°C throughout the analysis.

### Analysis of brush-border enzyme activity

Mucosal samples were taken from the proximal, middle, and distal sections of the jejunum, transferred into cryo-samples, which were then frozen at −80⁰C until the determination of brush-border enzyme activity. The activity of saccharase, lactase, and maltase in the brushstroke of the jejunum was determined according to a modified method of Dahlquist (1964 [75]). Briefly, the intestinal epithelium collected at postmortem was weighed, diluted in distilled water (1:4 v/v), and thoroughly homogenized using a mechanical homogenizer. The tissues were kept on ice at all times. The homogenate samples were then mixed with a solution containing substrate (sucrose, lactose, or maltose, respectively) and maleic acid reaction buffer. The samples were incubated at 37 °C for 1 h, and then an inhibitory solution containing Tris was immediately added to stop disaccharidase activity. At the same time, blank samples were prepared, where the inhibition solution was immediately added to the mixture of homogenates, substrate, and reaction buffer. Samples prepared in this way were applied to a microplate. In the next step, a commercially available Glucose RTU reagent was added to the homogenate samples to determine glucose concentration. A standard curve was prepared using the included glucose standard, mixing it with the inhibition solution. The microplate was incubated for 15 min. at 37°C, followed by an instantaneous absorbance reading at 490 nm using a Shimadzu UV-VIS1900i spectrophotometer (Kyoto, Japan). To convert the enzymatic activity of the disaccharidases, the total protein content of the homogenates was determined using a commercial test and spectrophotometric method, following the manufacturer’s instructions.

### Determination of the activity of aminopeptidases A and N and dipeptidylpeptidase IV in the brush border

The activity of aminopeptidases A and N and dipeptidylpeptidase IV in the brushstroke of the jejunum was determined according to the modified method of Maroux et al. (1973 [76]). Briefly, intestinal epithelium collected at postmortem was weighed, diluted in distilled water (1:4 v/v) and thoroughly homogenized using a mechanical homogenizer. The tissues were kept on ice at all times. Enzyme activity was measured by spectrophotometry using synthetic substrates: l-glutamic acid pnitroanilide (GAN), leucine p-nitroanilide (L-p-NA) or glycyl-L-prolyl p-nitroanilide tosylate for aminopeptidase A, aminopeptidase N or dipeptidylpeptidase IV, respectively. The homogenates were mixed with the appropriate substrate and reaction buffer, and the reactions were carried out in cuvettes at 37 °C. The concentration of the final reaction product, para-nitroaniline, was determined by kinetic absorbance measurement at 410 nm on a Shimadzu UV-VIS1900i spectrometer (Kyoto, Japan). A solution of the substrate suitable for the enzyme was used as a blank. For conversion of enzyme activity, the total protein content of the homogenates was determined using a commercial assay and spectrophotometric method, following the manufacturer’s instructions.

### RNA isolation and quantitative PCR

RNA isolation was conducted using the Total RNA Mini (A&A Biotechnology, Gdańsk, Poland) according to the producer’s recommendations. The RNA quality was assessed using the Tapestation 2200 (Agilent, Santa Clara, CA, USA), while its quantity was measured with the Nanodrop 2200 (Thermofisher Scientific, Waltham, MA, USA). The RNA quality was assessed by agarose gel electrophoresis. The RNA was then reverse transcribed using the High-Capacity cDNA Archive Kit (Thermofisher Scientific, Waltham, MA, USA). Subsequently, qPCR was performed using TaqMan

Gene Expression Assays: OCLN Ss3377507_u1, IL6 Ss3384604_u1, MUC4 Ss04321844_m1, TGFB1 Ss04955543_m1, CLDN2Ss3337502_u1, TNF Ss03391318_g1, IL10 Ss03382372_u1, IL4 Ss33394125_m1, CLDN4 Ss03375006_u1, CLDN1Ss03375708_u1. The process was performed in triplicate on the QuantStudio 7-flex instrument (Thermofisher Scientific, Waltham, MA, USA) using the TaqMan Gene Expression Master Mix. For an endogenous control, RPL27 Ss3385714_g1 was used.

### Statistical analysis

Data on basic, hematological, biochemical, histological, and sugar absorption analysis parameters were presented as mean and standard error of measurement (SEM) and analyzed using one-way ANOVA, followed by Tukey’s post hoc test with Bonferroni correction for the honestly significant difference to adjust for multiple comparisons. Gene expression data, immunohistochemical, and brush border enzyme activities were analyzed using the Kruskal-Wallis test, followed by post-hoc tests. Statistical analyses were performed using Statistica® version 13.3 (StatSoft) and GraphPad Prism version 10.0.2 for Windows (GraphPad Software). A P-value of less than 0.05 was considered statistically significant. Each piglet served as an experimental unit (n = 6 per group). Normal distribution of the data was confirmed using the Shapiro–Wilk W test, and homogeneity of variances was verified using the Brown– Forsythe test.

## References

[1] Pluske JR, Turpin DL, Kim J-C. Gastrointestinal tract (gut) health in the young pig. Anim Nutr. 2018;4(2):187–96. doi: 10.1016/j.aninu.2017.12.004 30140758 PMC6104527

[2] Tang X, Xiong K, Fang R, Li M. Weaning stress and intestinal health of piglets: A review. Front Immunol. 2022;13:1042778. doi: 10.3389/fimmu.2022.1042778 36505434 PMC9730250

[3] Tang X, Liu H, Yang S, Li Z, Zhong J, Fang R. Epidermal Growth Factor and Intestinal Barrier Function. Mediators Inflamm. 2016;2016:1927348. doi: 10.1155/2016/1927348 27524860 PMC4976184

[4] Hansen CF, Thymann T, Andersen AD, Holst JJ, Hartmann B, Hilsted L, et al. Rapid gut growth but persistent delay in digestive function in the postnatal period of preterm pigs. Am J Physiol Gastrointest Liver Physiol. 2016;310(8):G550-60. doi: 10.1152/ajpgi.00221.2015 26822913 PMC4836131

[5] Upadhaya S-D, Kim I-H. The Impact of Weaning Stress on Gut Health and the Mechanistic Aspects of Several Feed Additives Contributing to Improved Gut Health Function in Weanling Piglets-A Review. Animals (Basel). 2021;11(8):2418. doi: 10.3390/ani11082418 34438875 PMC8388735

[6] Ma TY, Nighot P, Al-Sadi R. Tight Junctions and the Intestinal Barrier. Physiology of the Gastrointestinal Tract. Elsevier. 2018. p. 587–639. doi: 10.1016/b978-0-12-809954-4.00025-6

[7] Moonwiriyakit A, Pathomthongtaweechai N, Steinhagen PR, Chantawichitwong P, Satianrapapong W, Pongkorpsakol P. Tight junctions: from molecules to gastrointestinal diseases. Tissue Barriers. 2023;11(2):2077620. doi: 10.1080/21688370.2022.2077620 35621376 PMC10161963

[8] Edelblum KL, Turner JR. The tight junction in inflammatory disease: communication breakdown. Curr Opin Pharmacol. 2009;9(6):715–20. doi: 10.1016/j.coph.2009.06.022 19632896 PMC2788114

[9] Arnott I, Ghosh S, Ferguson A. Abnormal intestinal permeability predicts relapse in patients with inactive Crohn’s disease. Gastroenterology. 1998;114:A923. doi: 10.1016/s0016-5085(98)83760-8 11145287

[10] König J, Wells J, Cani PD, García-Ródenas CL, MacDonald T, Mercenier A, et al. Human Intestinal Barrier Function in Health and Disease. Clin Transl Gastroenterol. 2016;7(10):e196. doi: 10.1038/ctg.2016.54 27763627 PMC5288588

[11] Neurath MF, Artis D, Becker C. The intestinal barrier: a pivotal role in health, inflammation, and cancer. Lancet Gastroenterol Hepatol. 2025;10(6):573–92. doi: 10.1016/S2468-1253(24)00390-X 40086468

[12] Bischoff SC, Barbara G, Buurman W, Ockhuizen T, Schulzke J-D, Serino M, et al. Intestinal permeability--a new target for disease prevention and therapy. BMC Gastroenterol. 2014;14:189. doi: 10.1186/s12876-014-0189-7 25407511 PMC4253991

[13] Modina SC, Polito U, Rossi R, Corino C, Di Giancamillo A. Nutritional Regulation of Gut Barrier Integrity in Weaning Piglets. Animals (Basel). 2019;9(12):1045. doi: 10.3390/ani9121045 31795348 PMC6940750

[14] Salminen S, Isolauri E, Salminen E. Clinical uses of probiotics for stabilizing the gut mucosal barrier: successful strains and future challenges. Antonie Van Leeuwenhoek. 1996;70(2–4):347–58. doi: 10.1007/BF00395941 8992950

[15] Khoshbin K, Camilleri M. Effects of dietary components on intestinal permeability in health and disease. Am J Physiol Gastrointest Liver Physiol. 2020;319(5):G589–608. doi: 10.1152/ajpgi.00245.2020 32902315 PMC8087346

[16] Dodd D, Spitzer MH, Van Treuren W, Merrill BD, Hryckowian AJ, Higginbottom SK, et al. A gut bacterial pathway metabolizes aromatic amino acids into nine circulating metabolites. Nature. 2017;551(7682):648–52. doi: 10.1038/nature24661 29168502 PMC5850949

[17] Sangild PT, Siggers RH, Schmidt M, Elnif J, Bjornvad CR, Thymann T, et al. Diet- and colonization-dependent intestinal dysfunction predisposes to necrotizing enterocolitis in preterm pigs. Gastroenterology. 2006;130(6):1776–92. doi: 10.1053/j.gastro.2006.02.026 16697741

[18] van Barneveld RJ, Dunshea FR. Colostrum Protein Isolate Increases Gut and Whole Body Growth and Plasma IGF-I in Neonatal Pigs. Asian Australas J Anim Sci. 2011;24(5):670–7. doi: 10.5713/ajas.2011.90490

[19] Playford RJ, Weiser MJ. Bovine Colostrum: Its Constituents and Uses. Nutrients. 2021;13(1):265. doi: 10.3390/nu13010265 33477653 PMC7831509

[20] Thapa BR. Health factors in colostrum. Indian J Pediatr. 2005;72(7):579–81. doi: 10.1007/BF02724182 16077241

[21] Field CJ. The immunological components of human milk and their effect on immune development in infants. J Nutr. 2005;135(1):1–4. doi: 10.1093/jn/135.1.1 15623823

[22] Shah NP. Effects of milk-derived bioactives: an overview. Br J Nutr. 2000;84 Suppl 1:S3-10. doi: 10.1017/s000711450000218x 11242440

[23] Silva SV, Malcata FX. Caseins as source of bioactive peptides. International Dairy Journal. 2005;15(1):1–15. doi: 10.1016/j.idairyj.2004.04.009

[24] Liepke C, Adermann K, Raida M, Mägert H-J, Forssmann W-G, Zucht H-D. Human milk provides peptides highly stimulating the growth of bifidobacteria. Eur J Biochem. 2002;269(2):712–8. doi: 10.1046/j.0014-2956.2001.02712.x 11856332

[25] Maciag SS, Bellaver FV, Bombassaro G, Haach V, Morés MAZ, Baron LF, et al. On the influence of the source of porcine colostrum in the development of early immune ontogeny in piglets. Sci Rep. 2022;12(1):15630. doi: 10.1038/s41598-022-20082-1 36115917 PMC9482628

[26] Donohoe DR, Garge N, Zhang X, Sun W, O’Connell TM, Bunger MK, et al. The microbiome and butyrate regulate energy metabolism and autophagy in the mammalian colon. Cell Metab. 2011;13(5):517–26. doi: 10.1016/j.cmet.2011.02.018 21531334 PMC3099420

[27] Wang H-B, Wang P-Y, Wang X, Wan Y-L, Liu Y-C. Butyrate enhances intestinal epithelial barrier function via up-regulation of tight junction protein Claudin-1 transcription. Dig Dis Sci. 2012;57(12):3126–35. doi: 10.1007/s10620-012-2259-4 22684624

[28] Zheng L, Kelly CJ, Battista KD, Schaefer R, Lanis JM, Alexeev EE, et al. Microbial-Derived Butyrate Promotes Epithelial Barrier Function through IL-10 Receptor-Dependent Repression of Claudin-2. J Immunol. 2017;199(8):2976–84. doi: 10.4049/jimmunol.1700105 28893958 PMC5636678

[29] Winnicka A. Wartości referencyjne podstawowych badań laboratoryjnych w weterynarii. Warszawa: Wydawnictwo SGGW. 2021.

[30] Turner JR. Intestinal mucosal barrier function in health and disease. Nat Rev Immunol. 2009;9(11):799–809. doi: 10.1038/nri2653 19855405

[31] Helander HF, Fändriks L. Surface area of the digestive tract - revisited. Scand J Gastroenterol. 2014;49(6):681–9. doi: 10.3109/00365521.2014.898326 24694282

[32] Salvo Romero E, Alonso Cotoner C, Pardo Camacho C, Casado Bedmar M, Vicario M. The intestinal barrier function and its involvement in digestive disease. Rev Esp Enferm Dig. 2015;107(11):686–96. doi: 10.17235/reed.2015.3846/2015 26541659

[33] Pieszka M, Szczepanik K, Łoniewski I. Utilizing Pigs as a Model for Studying Intestinal Barrier Function – A Review. Annals of Animal Science. 2025;25(2):569–83. doi: 10.2478/aoas-2024-0094

[34] Blecha F, Pollmann DS, Nichols DA. Weaning pigs at an early age decreases cellular immunity. J Anim Sci. 1983;56(2):396–400. doi: 10.2527/jas1983.562396x 6841290

[35] Yu K, Canalias F, Solà-Oriol D, Arroyo L, Pato R, Saco Y, et al. Age-Related Serum Biochemical Reference Intervals Established for Unweaned Calves and Piglets in the Post-weaning Period. Front Vet Sci. 2019;6:123. doi: 10.3389/fvets.2019.00123 31069239 PMC6491529

[36] Rymut HE, Rund LA, Bolt CR, Villamil MB, Bender DE, Southey BR, et al. Biochemistry and Immune Biomarkers Indicate Interacting Effects of Pre- and Postnatal Stressors in Pigs across Sexes. Animals (Basel). 2021;11(4):987. doi: 10.3390/ani11040987 33915976 PMC8067328

[37] Luo Z, Zhu W, Guo Q, Luo W, Zhang J, Xu W, et al. Weaning Induced Hepatic Oxidative Stress, Apoptosis, and Aminotransferases through MAPK Signaling Pathways in Piglets. Oxid Med Cell Longev. 2016;2016:4768541. doi: 10.1155/2016/4768541 27807471 PMC5078666

[38] Przybylska J, Albera E, Kankofer M. Antioxidants in bovine colostrum. Reprod Domest Anim. 2007;42(4):402–9. doi: 10.1111/j.1439-0531.2006.00799.x 17635778

[39] Yalçıntaş YM, Duman H, López JMM, Portocarrero ACM, Lombardo M, Khallouki F, et al. Revealing the Potency of Growth Factors in Bovine Colostrum. Nutrients. 2024;16(14):2359. doi: 10.3390/nu16142359 39064802 PMC11279796

[40] Chapter 5 Bioactive compounds in porcine colostrum and milk and their effects on intestinal development in neonatal pigs. Biology of Growing Animals. Elsevier. 2002. p. 169–92. doi: 10.1016/s1877-1823(09)70121-3

[41] Moeser AJ, Klok CV, Ryan KA, Wooten JG, Little D, Cook VL, et al. Stress signaling pathways activated by weaning mediate intestinal dysfunction in the pig. Am J Physiol Gastrointest Liver Physiol. 2007;292(1):G173-81. doi: 10.1152/ajpgi.00197.2006 16901995

[42] Vancamelbeke M, Vermeire S. The intestinal barrier: a fundamental role in health and disease. Expert Rev Gastroenterol Hepatol. 2017;11(9):821–34. doi: 10.1080/17474124.2017.1343143 28650209 PMC6104804

[43] Guo R, Jiang C, Niu Y, Niu C, Chen B, Yuan Z, et al. ShenQiGan Extract Repairs Intestinal Barrier in Weaning-Stressed Piglets by Modulating Inflammatory Factors, Immunoglobulins, and Short-Chain Fatty Acids. Animals (Basel). 2025;15(15):2218. doi: 10.3390/ani15152218 40805008 PMC12345433

[44] Rasmussen SO, Martin L, Østergaard MV, Rudloff S, Li Y, Roggenbuck M, et al. Bovine colostrum improves neonatal growth, digestive function, and gut immunity relative to donor human milk and infant formula in preterm pigs. Am J Physiol Gastrointest Liver Physiol. 2016;311(3):G480-91. doi: 10.1152/ajpgi.00139.2016 27445345

[45] Mei J, Zhang Y, Wang T, Sangild PT, Xu R-J. Oral ingestion of colostrum alters intestinal transforming growth factor-beta receptor intensity in newborn pigs. Livestock Science. 2006;105(1–3):214–22. doi: 10.1016/j.livsci.2006.06.017

[46] Biagi G, Piva A, Moschini M, Vezzali E, Roth FX. Performance, intestinal microflora, and wall morphology of weanling pigs fed sodium butyrate. J Anim Sci. 2007;85(5):1184–91. doi: 10.2527/jas.2006-378 17296766

[47] Kotunia A, W J, L D, J M, R V, G P, et al. Effect of sodium butyrate on the small intestine. Journal of Physiology and Pharmacology. 2004;:59–68. 15608361

[48] Wang JF, CYX, WZX, DSH, LZW. Effect of sodium butyrate on the structure of the small intestine mucous epithelium of weaning piglets. Chinese Journal of Veterinary Science and Technology. 2005;35:298–301.

[49] Lu J, Zou X, Wang Y. Effects of sodium butyrate on the growth performance, intestinal microflora and morphology of weanling pigs. J Anim Feed Sci. 2008;17(4):568–78. doi: 10.22358/jafs/66685/2008

[50] Roselli M, Britti MS, Le Huërou-Luron I, Marfaing H, Zhu WY, Mengheri E. Effect of different plant extracts and natural substances (PENS) against membrane damage induced by enterotoxigenic Escherichia coli K88 in pig intestinal cells. Toxicol In Vitro. 2007;21(2):224–9. doi: 10.1016/j.tiv.2006.09.012 17084584

[51] Solomons NW. Modulation of the immune system and the response against pathogens with bovine colostrum concentrates. Eur J Clin Nutr. 2002;56 Suppl 3:S24-8. doi: 10.1038/sj.ejcn.1601480 12142957

[52] Gopal PK, Gill HS. Oligosaccharides and glycoconjugates in bovine milk and colostrum. Br J Nutr. 2000;84 Suppl 1:S69-74. doi: 10.1017/s0007114500002270 11242449

[53] Shigetomi K, Ikenouchi J. Regulation of the epithelial barrier by post-translational modifications of tight junction membrane proteins. J Biochem. 2018;163(4):265–72. doi: 10.1093/jb/mvx077 29186552

[54] Zhao X, Zeng H, Lei L, Tong X, Yang L, Yang Y, et al. Tight junctions and their regulation by non-coding RNAs. Int J Biol Sci. 2021;17(3):712–27. doi: 10.7150/ijbs.45885 33767583 PMC7975691

[55] Sciascia QL, Metges CC. Review: Methods and biomarkers to investigate intestinal function and health in pigs. Animal. 2023;17 Suppl 3:100860. doi: 10.1016/j.animal.2023.100860 37316380

[56] Kalach N, Rocchiccioli F, de Boissieu D, Benhamou P, Dupont C. Intestinal permeability in children: variation with age and reliability in the diagnosis of cow’s milk allergy. Acta Paediatrica. 2001;90(5):499–504. doi: 10.1111/j.1651-2227.2001.tb00788.x 11430707

[57] Boudry G, Yang P-C, Perdue MH. Small intestine; Anatomy. Encyclopedia of Gastroenterology. Elsevier. 2004. p. 482–6. doi: 10.1016/b978-0-12-812460-4.00648-0

[58] Sadurní M, Barroeta AC, Sol C, Puyalto M, Castillejos L. Effects of dietary crude protein level and sodium butyrate protected by medium-chain fatty acid salts on performance and gut health in weaned piglets. J Anim Sci. 2023;101:skad090. doi: 10.1093/jas/skad090 36967519 PMC10103067

[59] Hedemann MS, Bach Knudsen KE. Resistant starch for weaning pigs — Effect on concentration of short chain fatty acids in digesta and intestinal morphology. Livestock Science. 2007;108(1–3):175–7. doi: 10.1016/j.livsci.2007.01.045

[60] Samuelsen AB. The traditional uses, chemical constituents and biological activities of Plantago major L. A review. J Ethnopharmacol. 2000;71(1–2):1–21. doi: 10.1016/s0378-8741(00)00212-9 10904143 PMC7142308

[61] Canani RB, Costanzo MD, Leone L, Pedata M, Meli R, Calignano A. Potential beneficial effects of butyrate in intestinal and extraintestinal diseases. World J Gastroenterol. 2011;17(12):1519–28. doi: 10.3748/wjg.v17.i12.1519 21472114 PMC3070119

[62] Leonel AJ, Alvarez-Leite JI. Butyrate: implications for intestinal function. Curr Opin Clin Nutr Metab Care. 2012;15(5):474–9. doi: 10.1097/MCO.0b013e32835665fa 22797568

[63] Soret R, Chevalier J, De Coppet P, Poupeau G, Derkinderen P, Segain JP, et al. Short-chain fatty acids regulate the enteric neurons and control gastrointestinal motility in rats. Gastroenterology. 2010;138(5):1772–82. doi: 10.1053/j.gastro.2010.01.053 20152836

[64] Bajka BH, Clarke JM, Topping DL, Cobiac L, Abeywardena MY, Patten GS. Butyrylated starch increases large bowel butyrate levels and lowers colonic smooth muscle contractility in rats. Nutr Res. 2010;30(6):427–34. doi: 10.1016/j.nutres.2010.06.003 20650351

[65] Cuche G, Malbert CH. Short-chain fatty acids present in the ileum inhibit fasting gastrointestinal motility in conscious pigs. Neurogastroenterol Motil. 1999;11(3):219–25. doi: 10.1046/j.1365-2982.1999.00149.x 10354346

[66] Kelly D, King TP, McFadyen M, Travis AJ. Effect of lactation on the decline of brush border lactase activity in neonatal pigs. Gut. 1991;32(4):386–92. doi: 10.1136/gut.32.4.386 1902807 PMC1379076

[67] Marion J, Petersen YM, Romé V, Thomas F, Sangild PT, Le Dividich J, et al. Early weaning stimulates intestinal brush border enzyme activities in piglets, mainly at the posttranscriptional level. J Pediatr Gastroenterol Nutr. 2005;41(4):401–10. doi: 10.1097/01.mpg.0000177704.99786.07 16205506

[68] Pluske JR, Kerton DK, Cranwell PD, Campbell RG, Mullan BP, King RH, et al. Age, sex, and weight at weaning influence organ weight and gastrointestinal development of weanling pigs. Australian Journal of Agricultural Research. 2003;54(5):515–27. doi: 10.1071/ar02156

[69] HAMPSON DJ, KIDDER DE. Influence of creep feeding and weaning on brush border enzyme activities in the piglet small intestine. Research in Veterinary Science. 1986;40(1):24–31. doi: 10.1016/s0034-5288(18)30481-8 3085180

[70] Woliński J, Słupecka M, Weström B, Prykhodko O, Ochniewicz P, Arciszewski M, et al. Effect of feeding colostrum versus exogenous immunoglobulin G on gastrointestinal structure and enteric nervous system in newborn pigs. J Anim Sci. 2012;90 Suppl 4:327–30. doi: 10.2527/jas.53926 23365369

[71] Chen L-H, Canibe N, Curtasu MV, Hedemann MS. Untargeted metabolomics as a tool to assess the impact of dietary approaches on pig gut health: a review. J Anim Sci Biotechnol. 2025;16(1):106. doi: 10.1186/s40104-025-01238-1 40691647 PMC12281987

[72] Grela E, SJ. Zalecenia żywieniowe i wartość pokarmowa pasz dla świń: monografia. The Kielanowski Institute of Animal Physiology and Nutrition, Polish Academy of Sciences. 2020.

[73] Bach Knudsen KE, Lærke HN, Hedemann MS, Nielsen TS, Ingerslev AK, Gundelund Nielsen DS, et al. Impact of Diet-Modulated Butyrate Production on Intestinal Barrier Function and Inflammation. Nutrients. 2018;10(10):1499. doi: 10.3390/nu10101499 30322146 PMC6213552

[74] Boudry C, Dehoux J-P, Wavreille J, Portetelle D, Théwis A, Buldgen A. Effect of a bovine colostrum whey supplementation on growth performance, faecal Escherichia coli population and systemic immune response of piglets at weaning. Animal. 2008;2(5):730–7. doi: 10.1017/S175173110800164X 22443598

[75] Dahlqvist A. Method for assay of intestinal disaccharidases. Anal Biochem. 1964;7:18–25. doi: 10.1016/0003-2697(64)90115-0 14106916

[76] Maroux S, Louvard D, Baratti J. The aminopeptidase from hog intestinal brush border. Biochim Biophys Acta. 1973;321(1):282–95. doi: 10.1016/0005-2744(73)90083-1 4750768

